# Validation of a HILIC UHPLC-MS/MS Method for Amino Acid Profiling in Triticum Species Wheat Flours

**DOI:** 10.3390/foods8100514

**Published:** 2019-10-18

**Authors:** Emmanouil Tsochatzis, Maria Papageorgiou, Stavros Kalogiannis

**Affiliations:** 1 Department of Chemical Engineering, Aristotle University of Thessaloniki, GR-54124 Thessaloniki, Greece; 2 Department of Food Science and Technology, International Hellenic University, Sindos Campus, P.O. Box 141, GR-57400 Thessaloniki, Greece; mariapapage@food.teithe.gr; 3 Department of Nutritional Sciences and Dietetics, International Hellenic University, Sindos Campus, P.O. Box 141, GR-57400 Thessaloniki, Greece; kalogian@nutr.teithe.gr

**Keywords:** amino acid profiling, hydrophilic interaction chromatography (HILIC), tandem mass spectrometry, Triticum species flours, flour quality characteristics

## Abstract

Amino acids are essential nutritional components as they occur in foods either in free form or as protein constituents. An ultra-high-performance (UHPLC) hydrophilic liquid chromatography (HILIC)-tandem Mass Spectrometry (MS) method has been developed and validated for the quantification of 17 amino acids (AA) in wheat flour samples after acid hydrolysis with 6 M HCl in the presence of 4% (*v*/*v*) thioglycolic acid as a reducing agent. The developed method proved to be a fast and reliable tool for acquiring information on the AA profile of cereal flours. The method has been applied and tested in 10 flour samples of spelt, emmer, and common wheat flours of organic or conventional cultivation and with different extraction rates (70%, 90%, and 100%). All the aforementioned allowed us to study and evaluate the variation of the AA profile among the studied flours, in relation to other quality characteristics, such as protein content, wet gluten, and gluten index. Significant differences were observed in the AA profiles of the studied flours. Moreover, AA profiles exhibited significant interactions with quality characteristics that proved to be affected based mainly on the type of grain. A statistical and multivariate analysis of the AA profiles and quality characteristics has been performed, as to identify potential interactions between protein content, amino acids, and quality characteristics.

## 1. Introduction

Amino acids (AA) are essential nutritional components present in foods either in their free form (FAA) or as protein constituents. They directly contribute to the flavor of foods as they are precursors of aroma compounds and colors formed by thermal or enzymatic reactions during production, processing, and storage of food. Hence, information on the profile and amount of free AA is highly needed in food science and nutrition studies [1,2,3,4,5].

Analysis of AA, in either free form or in protein profile, was highly challenging due to their structural and polarity differences. Ion exchange liquid chromatography has found wide application in AA analysis, especially through the commercial amino acid analyzers, which utilize cation-exchange chromatography followed by post-column derivatization with a chromophore or fluorophore derivatizing agent [6,7,8]. On the other hand, conventional reversed-phase (RP) High Pressure Liquid Chromatography (HPLC) proved to be time-consuming since most AAs are highly polar and cannot be determined without pre- or post-column derivatization [2,9,10]. Recently the advancement of hydrophilic interaction chromatography (HILIC) and new analytical columns provided alternative paths for the profiling of AA by liquid chromatography (LC). HILIC is generally known to enhance the sensitivity of electrospray ionization-mass spectrometry (ESI–MS) detection, and it is increasingly employed in the analysis of polar analysis in various matrices, [3,5,11,12,13,14,15].

So far, the application of HILIC-MS has found limited use in the analysis of AA in food samples. To our knowledge, three methods have been reported on the determination of FAA in liquid food matrices such as juice, beer, honey, or tea [3], ginkgo seeds [5], and fruits of Ziziphus jujuba [14]. Only recently, it was developed a HILIC-MS/MS method for the determination of either free AA or amino acids profile in high protein content food matrix (mussels) [15]. Moreover, the analytical methods that determine amino acid profile (TAA) were developed for the application in pure protein samples, such as collagen [16] or bovine serum albumin (BSA) and angiotensin I [13]. In all these methods, single ion monitoring-MS (SIM-MS) detection [5] or tandem MS [4,13] were applied. By using tandem MS detection, the separation of isobaric AA was feasible in most cases, whereas the use of single MS detection led to increased total analysis time since longer chromatographic runs were needed for the separation of all compounds. Prior to the analysis of amino acids, proteins need to be hydrolyzed in order to release their constituting AA. The most commonly applied method is hydrolysis by digestion with a strong inorganic acid [13,15,16,17].

Cereals are considered as one of the basic foods consumed by humans and animals. The carbohydrates they contain provide approximately 50% of the total daily calories, whereas the proteins one-third of the total protein need [17]. The composition of amino acids varies among the proteins of the different cereal grains or flours. Wheat (*Triticum* species) is the 3rd most-produced cereal worldwide. Wheat proteins are known to be low in some amino acids that are considered essential for the human diet, especially lysine (the most deficient amino acid) and threonine (the second limiting amino acid) [18,19,20,21]. On the other hand, they are rich in glutamine and proline (s), the functional amino acids in dough formation. Tetraploid Emmer wheat (*Triticum turgidum* species, dicoccum, genomes AABB), also known as emmer, faro or zea in different countries, is hulled wheat that differs from the domestic species on the fact that the ripened seed head of the wild species shatters and spreads the seed onto the ground while in the domesticated counterpart the seed head remains intact, making it easier for humans to harvest the grain. It is considered as the ancestor of bread wheat and durum wheat growing in the margin of the Mediterranean area [22]. Hexaploid spelt, *Triticum aestivum* variety spelta (genomes AABBDD) is also a hulled cereal grain with high resistance to environmental factors (diseases, stress) showing good yields under disadvantageous conditions [23]. It is suitable for organic farming and contributes to agro-diversity [24]. Spelt is becoming widely used in the growing natural food market. It has been reported that spelt protein content showed a great variation depending on the genotype [21,25,26], but it is higher than in common wheat [25,27]. The amino acid composition of the proteins from spelt differs slightly from one of the modern bread and pasta wheats [21]. It has been suggested that spelt-based products could be potentially more digestible than those from common wheat. Certain ancient wheats, einkorn, spelt, emmer, and Khorasan are currently of particular interest for use in selected bakery products [21]. The development of new cultivars has been attempted with the aim to improve the content of all essential amino acids [17,25].

In the present work a single HILIC-MS/MS method was developed, validated and applied for the determination of 17 amino acids, in a comparative study for the determination of the TAA of ‘ancient’ wheat species such as emmer, spelt, and bread wheat (*Triticum aestivum*) with different extraction rates and cultivated under different practices (organic or conventional) in a fast and reliable way without the need of derivatization, preceded only by a simple hydrolysis procedure. Finally, analysis of variances and multivariate analysis were performed in order to explore potential interactions of the TAA with flour quality characteristics of samples under investigation.

## 2. Materials and Methods

### 2.1. Chemicals

Standards of amino acids (AA) namely phenylalanine (Phe), tryptophan (Trp), isoleucine (Ile), leucine (Leu), asparagine (Asn), methionine (Met), valine (Val), proline (Pro), tyrosine (Tyr), alanine (Ala), threonine (Thr), glycine (Gly), serine (Ser), glutamic acid (Glu), aspartic acid (Asp), arginine (Arg), glutamine (Gln), lysine (Lys), histidine (His), cysteine (Cys) and cystine (Cys2),were purchased from Sigma-Aldrich (Steinheim, Germany). Acetonitrile (ACN) and water (LC-MS grade) was purchased from Carlo Erba (Milan, Italy). Formic acid (LC-MS additive), TGA (thioglycolic acid; ≥98%), and ammonium formate (NH_4_HCO_2_) were purchased from Sigma-Aldrich (Steinheim, Germany). Analytical grade hydrochloric acid (HCl) 37% w/w was also supplied from Carlo Erba (Milan, Italy). The chromatographic column HILIC amide BEH, Acquity UPLC 1.7 m, 2 × 150 mm (Waters) were used.

### 2.2. Standard Solutions

Stock solutions of the compounds were prepared in 0.1 M HCl at a concentration of 10 mg mL^−1^ and stored in amber vials at −20 °C. Working standards were prepared from the stock solutions by appropriate dilution with ACN/water 95:5 (*v*/*v*) and stored at −20 °C.

### 2.3. Sample Preparation for Amino Acid Profile Analysis

Ten (10) Triticum flours were selected from the Greek market, to be tested for their AA concentration and quality characteristics. The commercial flour samples differed in their extraction rate, type of cultivation (organic or conventional), and type of wheat, as shown in Table 1. The first letter of codification corresponds to wheat type (‘S’ for spelt, ‘B’ for bread wheat and ‘E’ for Emmer) followed by a 2-digit number revealing the flour extraction rate and the character ‘O’ in the case of organically cultivated wheat.

An amount of 10 g of flour was selected from 4 different positions of the package and mixed properly to create a homogeneous material that was then kept in a chemical dryer until analysis. The remaining original sample was kept at 2–4 °C. A modified method reported previously from Tsochatzis et al. [15] has been applied for the determination of amino acid mass fraction (g/100 g protein) in the aforementioned homogenized flour samples. In brief, for the AA profile 10.0 ± 0.5 mg of dried flour was placed in a hydrolysis tube along with 100 μL of 6 M HCl containing 4% (*v*/*v*) TGA as the reducing agent. The tube was flushed with N_2_ gas to establish oxygen-free conditions, sealed, and heated at 110 °C for 18 h. Then, the mixture was transferred to a centrifuge tube with 0.5 mL HCl 0.1 M and 0.5 mL water and centrifuged at 4200× *g* for 5 min. The supernatant was collected and filtered through a 0.22 μm polytetrafluoroethylene (PTFE) syringe filter. An amount of 0.5 mL of the clear extract was diluted with 4.5 mL ACN/water 95:5 *v*/*v* and injected to the UHPLC-HILIC-ESI MS/MS system.

### 2.4. UHPLC-MS/MS Analysis

UHPLC–tandem mass spectrometry was based on the method described by Tsochatzis et al. [15]. In brief, the analysis was performed on an Accela TSQ Quantum TM Access MAX Triple Quadrupole Mass Spectrometer system (Thermo Scientific, San Jose, CA, USA) operating under XCalibur (Thermo Scientific, San Jose, CA, USA) Software. The mobile phase consisted of solvent A: ACN/5 mM HCOONH_4_, pH = 3.0 adjusted with HCOOH 95:5 (*v*/*v*) and solvent B: ACN/5 mM HCOONH_4_, pH = 3.0 adjusted with HCOOH, 40:60 (*v*/*v*). Elution was based on a linear gradient program of 13 min from 80% A:20% B to 62% A:38% B, followed by a 2 min equilibration step to the initial conditions prior to the next injection. The flow rate was 400 μL min^−1^, and the total analysis time was 15 min.

Chromatographic separation was performed on a 2.1 mm × 150 mm ACQUITY UPLC 1.7 μm BEH HILIC amide column (Waters), equipped with an ACQUITY UPLC BEH Amide 1.7 μm Van-Guard Pre-column, maintained at 40 °C. Selected Reaction Monitoring (SRM) with Electrospray positive ionization mode (ESI +) was applied with spray voltage at 3000 V, capillary temperature: 300 °C, vaporizer temperature: 300 °C, sheath gas pressure at 40 arbitrary units (Arb), aux gas pressure at 10 Arb, ion sweep gas pressure at 2.0 Arb, ion source discharge current at 4.0 μA and collision gas pressure at 1.5 mTorr. Auto-samplers’ temperature was set at 4 °C, and the injection volume set at 5 μL. Amino acid individual data regarding molecular formulas, monoisotopic masses, precursor-product ion for the aforementioned SRM, along with their respective retention times in standard solutions and after acidic hydrolysis, are given in the Supplementary material (Table S1).

### 2.5. Method Validation

Method linearity, precision, trueness, the limit of detection (LOD), and limit of quantification (LOQ) were calculated. The linearity of the method was firstly assessed by analyzing standard solutions mixtures at six concentration levels for all AA (0.5, 1, 10, 50, 100, 200 μg mL^−1^), representing the working concentration range. Calibration curves were constructed by plotting the peak areas of the respective AAs followed by linear regression analysis (R^2^), based on the standard addition method. LODs and LOQs were calculated according to the signal-to-noise ratio (S/N) and the slope (S), using the equations LOD = 3 SD/S and LOQ = 10 SD/S [28].

Precision and trueness were assessed in the B-90 flour sample, which was used as a reference sample. The sample was fortified at two concentration levels (20.0 and 40.0 mg/100 g) of all AAs tested. All calculations were performed using the concentration values expressed in g/100 g for each AA individually. For short-term repeatability, the fortified samples were analyzed in triplicates during the day while for intermediate precision, the aforementioned samples were analyzed in triplicates for three consecutive days. Relative standard deviation (RSD; %) and recoveries were calculated as ((amount found in the spiked sample—amount found in the sample)/amount added) × 100 [2]. All the results regarding precision and trueness are presented in Supplementary material’s Table S2.

### 2.6. Quantitation and Matrix Effect

The calibration curves of the studied AAs, based on the linear regression coefficients (R^2^) have been performed with the standard addition method. Flour samples were fortified at two concentration (5 and 10 μmol/100 g) levels of all AAs tested, followed by an analysis in triplicates. Calibration curves were constructed by linear regression analysis of the peak area (Y) versus the injected concentration (X), and they have been assessed based on the linear regression coefficients (R^2^). Linear equations were established to determine the initial concentration of amino acids in the dried cereal flour samples. Evaluation of the matrix effect (ME, %) was performed by the slope comparison method as it was previously reported [2,15].

### 2.7. Protein Content and Flour Quality Parameters

The moisture, ash, total protein content, wet gluten, and gluten index (GI) were determined following ΙCC standard methods 109/1, 104/1, 105/2, and 155, respectively [29].

### 2.8. Data Processing and Statistical Analysis

Data were processed using the XCalibur application manager for the quantification of compounds. Regression analysis and statistics were performed using Microsoft Excel, and further statistical analysis, such as Analysis of Variance (ANOVA), followed by Tukey comparison test in all cases, has been performed with Minitab 18.0 statistical software (Minitab Inc., State College, PA, USA). The multivariate statistical analysis, combined with cluster analysis, has been performed with Simca 15 (Umetrics, Umea, Sweden).

## 3. Results

### 3.1. Acidic Hydrolysis and Antioxidant Agent

A properly performed hydrolysis is a prerequisite of a successful analysis regarding amino acid profiling in food matrices. The conditions selected were based according to the previously reported conditions by Fountoulakis et al. and the applied conditions in case of mussels from Tsochatzis et al. [15] or the ones reported by EZ Faast [30]. We selected the conditions of 110 °C for 18 h, in order to minimize (in combination with the antioxidant agent) degradation of specific amino acids, while we made a compromise in the recoveries of the more hydrophobic AA, such as valine and isoleucine and leucine [6,15,30]. In addition, the selection of this temperature was selected to minimize the potential cross-reactions of amino-acids with starch. The selection of the hydrolysis conditions was also based on the study of Tsochatzis et al. [15].

### 3.2. Analytical Method Development, Validation, and Optimization

**Figure 1:**
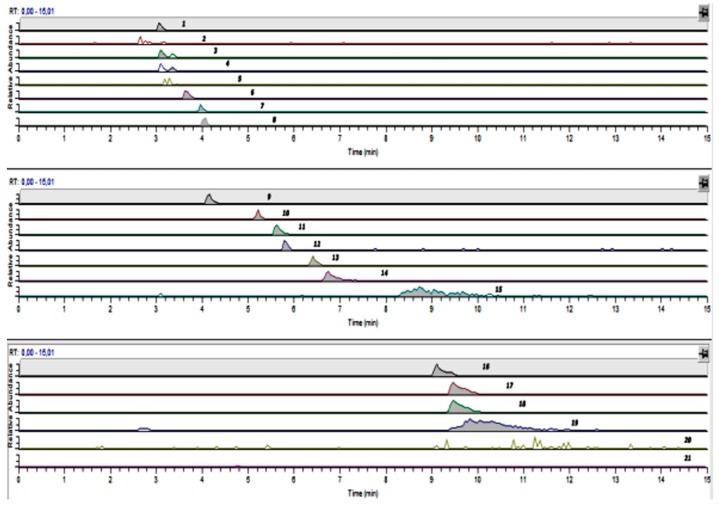
HILIC UHPLC–MS/MS chromatographic traces of the 17 amino acids quantified in the hydrolyzed flour sample B-90 (Elution order is 1: phenylalanine; 2: tryptophan; 3: Isoleucine; 4: leucine; 5: asparagine; 6: methionine; 7: valine; 8: proline; 9: tyrosine; 10: alanine; 11: threonine; 12: glycine; 13: serine; 14: glutamic acid; 15: aspartic acid; 16: arginine; 17: glutamine; 18: lysine; 19: histidine; 20: cystine; 21: cysteine).

The total analysis time was less than 12 min. The method exhibited good linearity in the concentration range of 0.5–200 μg mL^−1^ with a linear regression coefficient (R^2^) of above 0.99 for each AA. The effects from the matrix were minimal in both cases of either standard solutions or amino acid determination after hydrolysis. A typical chromatogram of the AA analysis in flours is presented in Figure 1.

The present analytical method exhibited satisfactory sensitivity for all AA. LODs varied from 0.002 (valine, serine, leucine, isoleucine) to 0.009 g/100 g (threonine) and the LOQs from 0.007 (serine, leucine) to 0.024 g/100 g (threonine, lysine) and a minimal matrix effect was observed. The analytical figures of merit of the method are given in Table 2.

Regarding the trueness, it was assessed by the recoveries from spiked cereal bread wheat flour, after acidic hydrolysis, at two concentration levels. The resulting recoveries ranged from 85.7% (lysine) to 121.8% (leucine) in the intra-day assay and from 86.8% (lysine) to 123.3% (leucine) for the intermediate precision (Table S2). The respective precision expressed in relative standard deviation (RSD %) values ranged from 0.6% (Glutamic acid) to 13.9% (proline) and from 1.4% (serine) to 13.7% (lysine) for short-term repeatability and intermediate precision respectively. The results of the analytical method presented adequate precision and accuracy results.

### 3.3. Amino Acid Profile of Flour Samples

Statistical differences have been identified between the AA mass fractions (Table 3) among the studied flour samples. From Table 3, it could be concluded that phenylalanine, threonine, glycine, and histidine did not have statistical differences among the various flours. Instead, significant differences have been observed for isoleucine, serine, lysine, valine, methionine, proline, glutamic acid, and glutamine. The results were in accordance with previously reported work, regarding the study of AA content in ancient cereal grain wheat cultivars [21].

### 3.4. Flour Quality Parameters

The results from all the studied quality characteristics of the flours are presented in Table 4. Gluten index and wet gluten have been evaluated, and ANOVA showed that the tested flours presented a significant difference with the” bread” wheat flour, also showed that flours do differ in their protein (%) content (data obtained from the Kjeldahl method), gluten index, and wet gluten. By comparing the set of ANOVA results, it could be identified that “bread” flours presented a large variety of total protein content, ranging from 12.3% (B-70) to 13.9 (B-90-O), while differences were also observed for their gluten index and wet gluten. In the case of spelta, the S-70 types, either organic or conventional, presented close results in all the studied quality characteristics, something which was also valid in case of “Emmer”-type.

## 4. Discussion

### 4.1. Acidic Hydrolysis

Regarding sample preparation and acidic hydrolysis, we studied the effect of the antioxidant agent as specific AA are susceptible to decomposition during acidic hydrolyses, such as tryptophan, as well as others like asparagine and glutamine to be converted to aspartic acid and glutamic acid, respectively. In this case, an antioxidant agent is needed to prevent the aforementioned decomposition or converting reactions. We selected to proceed with TGA, as it has been reported to be an effective antioxidant agent for amino acid analysis in food matrices [6,15,30]. In this case, the two levels of TGA have been tested, of 2% *v*/*v* [30] and 4% [15]. The results indicated that the presence of TGA at a level of 4% *v*/*v*, was more effective, and it was selected for the final protocol.

Even though it is a practice in acid hydrolysis to add an antioxidant agent to prevent the degradation of AAs, to our knowledge, such a protective step has not been previously applied prior to HILIC-MS analysis in case of cereal flour analysis. Moreover, no single set of conditions has been suggested so far for the effective prevention of all AA degradation in wheat flour samples.

### 4.2. Sample Preparation and Analytical Methods

By applying the present method, all AAs eluted prior to 11 min of run time. The selected chromatographic conditions, especially for the mobile phase, have been selected with the aim to provide as good peak shape for basic, neutral, and acidic AAs and certainly taking into consideration the effect of pH to HILIC peak shapes [15,31]. As reported previously by Tsochatzis et al., it was noticed that the retention times and the respective peak shapes of the analytes in the extracts are slightly shifted from the respective AA standard. This behavior could be due to the highly acidic conditions during hydrolysis, which affects the pH of the final injection sample and the separation of the analytes. Due to the contribution of ionic interactions in the retention mechanism, in HILIC, the charge state of the analyte affects the retention time of the compound [15,31]. To eliminate this effect, after trying different approaches, it was selected the dilution (10 times with ACN/water 95:5 *v*/*v* to obtain a final pH of 2.5–3) of the final solution before injection. The dilution did not present a limitation for the present method since the concentration of amino acids obtained after hydrolyzing the proteins is significantly higher than that of free amino acids present in most foods [15,32]. The Multiple Reaction Monitoring (MRM) transitions and the parameters in the tandem MS detection were selected after tuning for the optimum signal for each of the analytes.

Trueness assessed by the reported recoveries from spiked cereal flour after acidic hydrolysis. An important source of slight variations could be considered the acidic hydrolysis and especially the precision of the temperature that needs to be controlled and precisely assessed [32]. The reported results for both trueness and precision regarding the quantification of AAs are similar to the ones previously reported in other food matrices [3,5,14,15].

With the current method, only 17 amino acids could be determined following acid hydrolysis with TGA as an antioxidant agent. The amino acids tryptophan, cysteine, cystine, and asparagine were affected from the acid hydrolysis; tryptophan was unstable, asparagine and glutamine tended to convert to aspartic acid and glutamic acid, respectively, while cysteine and cystine were oxidized to cysteic acid [9,15,33].

### 4.3. Comparative Study of Amino Acid Profile of Flour Samples

Regarding stability and yields of amino-acids during acidic hydrolysis, serine and tyrosine are generated in low yields, methionine is sensitive to oxidation, due to acidic conditions it could be oxidized to its sulfone product, and finally, valine hydrolyzes in poor yields (longer time and temperatures are needed) [1,34,35].

In case of proline, Kapusniak et al. reported reactions of starch with α-amino acids, where proline, alanine, isoleucine, and valine most readily reacted with starch that could affect the hydrolysis [36] while, Ito et al. (2006) highlighted the significant effect of hydrolysis time on the yields of all amino acids, with changes in isoleucine, lysine and serine, while there was observed an existing binding of amino acids (glycine, alanine, and partial lysine) to starch chains [37].

In addition, partial losses of the amino acids tyrosine and serine while the other amino acids (valine, leucine, and isoleucine) are requiring longer hydrolysis time in order to obtain higher yields. Our results are in accordance with Rowan et al. [38].

It has been reported that spelt has a high concentration in methionine compared to wheat [25,39,40]. This fact was confirmed in the present study, where all tested spelt flours, presented significantly higher methionine content than emmer as well as bread wheat flours. The values of the methionine varied from 0.73–0.97 g/100 g protein (dry basis); spelt flours showed 10% higher values than the reference bread wheat flour. Aspartic acid content was significant higher in all studied spelt flours compared to whole grain wheat flour is in accordance with the results of [21,25,38].

In the case of alanine, the results indicated that all flours have significantly higher content compared to the B-90 except for E-100-O that showed similar value. The results suggest that hydrolysis has a great impact and effect on the release and analysis of the amino acids, while on the other hand, the side cultivation technique and location have a potential role in the final concentration of specific amino acids.

It is also reported in the literature that wheat proteins have low mass fractions of certain AA, especially lysine and threonine [18,19,20,21]. Contrary, in the current study, it was observed that the refined bread wheat flours and the whole grain wheat flour (reference) presented significantly higher contents of lysine (2.38 and 1.89 g/100 g protein) compared to other types of studied flours (emmer, spelt) that had significantly lower mass fractions [21]. It is also reported that wheat proteins have low mass fractions of certain AA, especially lysine and threonine [18,19,20,21].

Escarnot et al., reported that the spelt amino acid profile differs from that of bread wheat, supported by limited evidence of higher content for isoleucine, leucine, and glycine [25,40,41,42,43]. Our results are in accordance with the reported literature on higher protein content in spelt than in wheat grains under low nitrogen fertilization [25,40], although this has not been proved to be statistically significant, as it is also observed in our study. Pruska-Kedzior et al. found significantly higher protein content in spelt flour, but there should be considered that genotype and the cultivation conditions highly affect the protein content [27].

Statistical analysis (ANOVA) performed for each AA, indicated that there are significant statistical differences between all the studied flours (Table 3). Statistical analysis revealed some very interesting results about the interaction between the type of flour and the concentration of amino acids. In general, there was a significant interaction between the type of the flour studied and the amount of AA.

Glutamine and proline are the functional amino acids in dough formation [21]. For glutamine, the organic B-90 presented the higher mass fraction among the flours studied, followed by the S-70-O and conventional spelta S-100a. Especially in the case of glutamic acid (Glu) and proline, the whole grain wheat flour showed higher concentration than the B-90 (Table 3). White wheat flour and white whole grain flours showed lower proline and glutamic acid concentrations that the rest of flours studied, which is in accordance with the results of Abdel-Aal and Hucl and Escarnot et al. [21,25]. The aforementioned authors reported that the spelt is rich in proline, which is the major functional amino acid in dough formation. The data obtained showed that the organic bread wheat flour (B-90-O) showed much higher proline content than the two bread wheat flours B-70 and B-90).

### 4.4. Interaction with Quality Parameters

**Figure 2:**
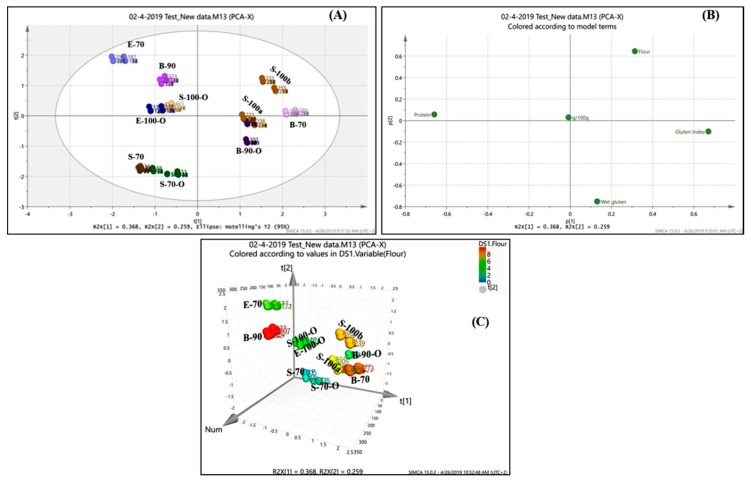
Principal components analysis of the studied amino acid profile and quality characteristics; (**A**) score plot and (**B**) loading plot and (**C**) 3D scatterplot (for the type studied cereal flour numbering, please see Table 1).

A multivariate analysis was performed to study potential interactions of the TAA with the quality characteristics of cereal flours under investigation. Thus, by developing this analytical tool that reveals the amino acid profile of flours, one could discover the amino acid profile of proteins of different Triticum species and use it for choosing the variety with a more balanced profile for the use in cereal product development in correlation with other quality characteristics. The performed multivariate analysis between the AA content and the flour quality characteristics showed that the studied cereal flours could be distinguished in groups, based on their origin. The score plot, along with the respective loadings is presented in Figure 2.

Results showed that the studied types of flours presented a specific AA content pattern and specific quality characteristics. From the principal component analysis (PCA) biplot and the respective score plots, it could be identified that there is a clear differentiation of the three different types of flours. Three different groups were identified as expected; the emmer grains (blue dots), the spelta (brown dots) and the bread wheat (purple dots). The right part represents the bread wheat flours, with high protein content (%), high gluten index, and lower falling number (FN). The Emmer type flours presented lower protein content and lower gluten index, while the spelt type flours are in between these two categories, presenting intermediate quality characteristics of the other two types of flours.

All the interactions and effects could be observed in the loading plot (Figure 2B). Briefly, the main effects resulted from the type of flour, and the protein content tends to play a significant factor in the clustering of the assessed grains which is resulting also to various quality characteristics and specific AA profiles, for which we have already identified statistical differences among the three types of flours. In addition, the organic cultivated grains tended to reach a higher protein content that is also reflecting to a higher content of certain AAs. On the other hand, a lower protein content reflected to a lower content of certain AA, but significant In concluding, it seems that the type of cultivation of the cereal grains affects the AA profile, as well as the quality characteristics of the flours and there is an indication of the potential effect of the cultivation (organic, conventional). Flours from organic cultivated grains seemed to be close in protein content (%) and eventually in the amino acids, but there is a clear differentiation in gluten index and wet gluten. In this case, the “spelt” flours are closer to “emmer” for the aforementioned characteristics, and all of them differentiated from the bread flour either type-70 or type-90. Spelt flours, either type-70 or 100, presented similar characteristics for either organic or conventional cultivated grains, while also “Emmer” type-70 or 100, presented also close characteristics. The biggest differentiation was noticed in the “bread” flours, where the cultivation and the extraction rate presented a significant effect for all the studied factors and the amino acid content (Table 3 and Figure 2).

## 5. Conclusions

A UHPLC-HILIC-tandem MS method has been developed and validated for the quantification of 17 amino acids in cereal flour samples after acid hydrolysis with HCl in the presence of a reducing agent. Tryptophan, cysteine, cystine, and asparagine were not possible to be quantified as they were degraded during hydrolysis due to harsh acidic conditions applied. The method proved to be a fast and reliable tool for acquiring information on amino acids profile from cereal flours. The developed analytical method has been applied in different flours such as spelt, dicoccum, whole grain wheat, and white wheat. Moreover, multivariate analysis showed that protein content and type of flour have the main contribution and effect, interacting with either the AA profile and with the studied quality characteristics. It has also been presented that not only with quality characteristics of the flours, but the type oz flour has significant interactions. A clear effect of an indication of the potential effect has been identified among the different flours studied.

## Figures and Tables

**Table 1:** Studied Triticum flours with their extraction rates, type of cultivation, and type of wheat.

No.	Extraction Rate	Cultivation	Wheat Type
1	70%	Conventional	Spelt (S-70)
2	70%	Organic	Spelt (S-70-O)
3	90%	Organic	Bread wheat (B-90-O)
4	100%	Organic	Emmer (E-100-O)
5	100%	Organic	Spelt (S-100-O)
6	70%	Conventional	Emmer (E-70)
7	100%	Conventional	Spelt (S-100a)
8	100%	Conventional	Spelt (S-100b)
9	70%	Conventional	Bread wheat (B-70)
10	90%	Conventional	Bread wheat (B-90)

**Table 2:** Limits of detection (LODs), limits of quantification (LOQs), linear ranges, and linear regression coefficients for the amino acids matrix match calibration in hydrolyzed cereal flour.

Amino Acid	Linear Regression (*R*^2^)	LOD (g/100 g)	LOQ (g/100 g)	Matrix Effect (%) *
Glycine	0.993	0.005	0.010	94.6
Alanine	0.994	0.007	0.020	106.4
Serine	0.996	0.002	0.007	88.1
Proline	0.999	0.004	0.013	107.8
Valine	0.995	0.002	0.007	111.7
Threonine	0.993	0.009	0.024	108.8
Aspartic acid	0.993	0.007	0.020	102.8
Glutamic acid	0.996	0.007	0.022	92.2
Isoleucine	0.999	0.002	0.009	82.6
Leucine	0.999	0.002	0.007	88.5
Asparagine	-			-
Lysine	0.993	0.009	0.024	82.2
Methionine	0.994	0.007	0.020	86.3
Histidine	0.994	0.007	0.022	83.6
Phenylalanine	0.996	0.007	0.020	85.3
Arginine	0.997	0.004	0.016	110.1
Tyrosine	0.997	0.004	0.016	109.2
Cystine	-	-	-	-
Cysteine	-	-	-	-

* Calculated based on the slope ratio method [2].

**Table 3:** Average amino acids composition (g/100 g protein) of wheat flours under investigation.

Target AA	Flour Sample (g/100 g Protein; Dry Basis) *									
	1	2	3	4	5	6	7	8	9	10
	Spelta (S-70)	Spelta (S-70-O)	Bread Wheat (B-90-O)	Emmer (E-100-O)	Spelta (S-100-O)	Emmer (E-70)	Spelta (S-100a)	Spelta (S-100b)	Bread Wheat (B-70)	Bread Wheat (B-90)
**Phe**	3.73 ^e^	3.94 ^d^	4.23 ^ab^	3.93 ^d^	3.99 ^cd^	3.52 ^g^	4.13 ^bc^	4.38 ^a^	3.71 ^ef^	3.54 ^fg^
**Ile**	1.59 ^cd^	1.75 ^a^	1.74 ^a^	1.55 ^de^	1.57 ^de^	1.50 ^ef^	1.70 ^ab^	1.65 ^bc^	1.32 ^g^	1.46 ^f^
**Leu**	3.82 ^d^	4.08 ^c^	4.18 ^bc^	4.03 ^c^	4.09 ^c^	3.76 ^d^	4.28 ^b^	4.49 ^a^	3.76 ^d^	3.46 ^e^
**Met**	0.96 ^a^	0.84 ^c^	0.91 ^b^	0.81 ^cd^	0.89 ^b^	0.81 ^cd^	0.88 ^b^	0.97 ^a^	0.79 ^d^	0.73 ^e^
**Tyr**	1.72 ^c^	1.88 ^ab^	1.95 ^a^	1.82 ^b^	1.84 ^b^	1.63 ^d^	1.83 ^b^	1.95 ^a^	1.65 ^cd^	1.64 ^d^
**Pro**	6.18 ^b^	6.87 ^a^	5.44 ^c^	4.44 ^ef^	4.64 ^de^	4.38 ^f^	4.69 ^d^	3.82 ^g^	2.97 ^i^	3.55 ^h^
**Val**	0.96 ^c^	1.10 ^a^	1.05 ^b^	0.87 ^d^	0.89 ^d^	0.81 ^e^	0.95 ^c^	0.82 ^e^	0.69 ^f^	0.73 ^f^
**Ala**	1.91 ^c^	1.94 ^c^	2.09 ^b^	1.68 ^d^	2.05 ^b^	1.88 ^c^	2.38 ^a^	1.12 ^f^	0.96 ^g^	1.52 ^e^
**Thr**	2.29 ^d^	2.59 ^a^	2.51 ^abc^	2.42 ^c^	2.46 ^bc^	2.13 ^e^	2.45 ^bc^	2.54 ^ab^	2.11 ^e^	1.86 ^f^
**Gly**	2.67 ^d^	2.07 ^e^	3.48 ^a^	2.02 ^e^	3.00 ^c^	2.13 ^e^	2.72 ^d^	3.44 ^a^	3.47 ^a^	3.15 ^b^
**Lys**	1.57 ^c^	1.43 ^de^	1.46 ^d^	1.28 ^f^	1.36 ^e^	1.25 ^f^	1.43 ^de^	1.42 ^de^	2.38 ^a^	1.89 ^b^
**Ser**	1.27 ^b^	1.43 ^a^	1.39 ^a^	1.28 ^b^	1.16 ^c^	1.25 ^b^	1.29 ^b^	1.12 ^cd^	0.86 ^e^	1.09 ^d^
**Arg**	4.68 ^e^	5.22 ^bc^	5.37 ^ab^	4.94 ^d^	5.02 ^cd^	4.60 ^ef^	4.99 ^d^	5.50 ^a^	4.39 ^f^	4.16 ^g^
**His**	2.42 ^c^	2.59 ^a^	2.65 ^a^	2.56 ^a^	2.59 ^a^	2.38 ^cd^	2.58 ^a^	2.54 ^ab^	2.44 ^bc^	2.30 ^d^
**Gln**	1.08 ^e^	1.30 ^ab^	1.32 ^a^	1.14 ^d^	1.23 ^c^	1.13 ^d^	1.22 ^c^	1.27 ^bc^	1.09 ^de^	1.09 ^de^
**Asp**	4.36 ^cd^	4.66 ^b^	5.02 ^a^	3.87 ^f^	4.42 ^c^	4.51 ^bc^	4.16 ^e^	4.04 ^ef^	4.18 ^de^	3.93 ^f^
**Glu**	41.33 ^e^	39.67^e^	41.47 ^e^	49.22 ^c^	52.41 ^ab^	50.10 ^c^	54.28 ^a^	44.83 ^d^	40.90 ^e^	41.25 ^bc^

* Values are means of triplicate measurements. Values followed by different letters within a row are significantly different (*p* < 0.05).

**Table 4:** Protein content of the tested flours.

No.	Wheat Type	Protein (% dw) *	Gluten Index	Wet Gluten
1	Spelt (S-70)	13.5 ^ab^	24 ^a^	37.4 ^d^
2	Spelt (S-70-O)	13.5 ^ab^	42 ^b^	39.8 ^d^
3	Bread wheat (B-90-O)	12.5 ^a^	94 ^c^	27.5 ^b^
4	Emmer (E-100-O)	13.1 ^ab^	19 ^a^	25.3 ^b^
5	Spelt (S-100-O)	12.9 ^ab^	20 ^a^	26.7 ^b^
6	Emmer (E-70)	14.1 ^c^	18 ^a^	15.1 ^a^
7	Spelta (S-100a)	12.0 ^a^	51 ^b^	33.3 ^c^
8	Spelta (S-100b)	12.0 ^a^	76 ^bc^	23.8 ^b^
9	Bread wheat (B-70)	12.3 ^a^	98 ^c^	33.8 ^c^
10	Bread wheat (B-90)	13.9 ^c^	27 ^a^	29.5 ^bc^

* Values followed by different letters within a column are significantly different (*p* < 0.05).

## Supplementary Materials

The following are available online at https://www.mdpi.com/2304-8158/8/10/514/s1, Table S1: Amino acids monoisotopic masses. Multiple Reaction Monitoring (MRM). Retention times (tR) and conditions in the mass spectrometer along with their respective molecular formulas, Table S2: Precision and trueness data for the analysis of AA in spiked B-90 bread wheat sample.
