# Supplementary material for: Validation of a HILIC UHPLC-MS/MS Method for Amino Acid Profiling in Triticum Species Wheat Flours
Source: Foods. 2019 Oct 18;8(10):514. doi: 10.3390/foods8100514 (PMC6836243; doi:10.3390/foods8100514)
Supplement: Supplementary file 1 [file foods-08-00514-s001.pdf]

Supplementary Material

Table S1. Amino acids monoisotopic masses. MRMs. retention times (t_R) and conditions in the mass spectrometer along with their respective molecular formulas.

Analyte	Molecular Formula	Monoisotopic mass	Precursor Ion	Product Ion	Collision Energy (V)	Tube Lens	t_R standard solutions (min) (Mean±S_d*)	t_R hydrolysed sample (min) (Mean ± S_d*)
Phe	$C_9H_{11}NO_2$	165.19	166.1	120.2	14	47	2.50 ± 0.06	3.12 ± 0.13
Trp	$C_{11}H_{12}N_2O_2$	204.23	205.1	188.1	12	52	2.58 ± 0.06	-
Ile	$C_6H_{13}NO_2$	131.17	132.1	86.3	6	40	2.67 ± 0.06	3.18 ± 0.15
Leu	$C_6H_{13}NO_2$	131.17	132.1	86.3	11	49	3.19 ± 0.07	3.38 ± 0.17
Asn	$C_4H_8N_2O_3$	132.12	133.1	87.2	8	49	2.74 ± 0.05	-
Met	$C_5H_{11}NO_2S$	149.21	150.1	56.4	17	46	3.15 ± 0.07	3.70 ± 0.12
Val	$C_5H_{11}NO_2$	117.15	118.1	72.3	11	46	3.22 ± 0.03	3.99 ± 0.14
Pro	$C_5H_9NO_2$	115.13	116.1	70.3	17	51	3.56 ± 0.07	4.02 ± 0.16
Tyr	$C_9H_{11}NO_3$	181.19	182.1	136.2	14	46	3.53 ± 0.05	4.24 ± 0.12
Ala	$C_3H_7NO_2$	89.094	90.1	44.5	10	40	4.55 ± 0.10	5.26 ± 0.11
Thr	$C_4H_9NO_3$	119.12	120.1	74.3	11	46	4.91 ± 0.03	5.73 ± 0.11
Gly	$C_2H_5NO_2$	75.07	76.1	30.6	12	40	5.22 ± 0.04	5.85 ± 0.15
Ser	$C_3H_7NO_3$	105.09	106.1	60.4	12	60	5.74 ± 0.07	6.47 ± 0.13
Glu	$C_5H_9NO_4$	147.13	148.0	84.0	15	70	5.94 ± 0.09	6.85 ± 0.11
Asp	$C_4H_7NO_4$	133.11	134.1	88.3	11	70	7.86 ± 0.12	8.81 ± 0.16
Arg	$C_6H_{14}N_4O_2$	174.20	175.1	70.3	23	54	8.35 ± 0.11	9.18 ± 0.11
Gln	$C_5H_{10}N_2O_3$	146.15	147.1	130.2	9	45	8.78 ± 0.12	9.54 ± 0.12
Lys	$C_6H_{14}N_2O_2$	146.11	147.1	84.3	17	48	8.79 ± 0.11	9.54 ± 0.14
His	$C_6H_9N_3O_2$	155.16	156.1	110.2	15	49	9.20 ± 0.10	9.92 ± 0.17
Cys_2	$C_6H_{12}N_2O_4S_2$	240.29	241.0	152.0	13	76	10.24 ± 0.08	-
Cys	$C_3H_7NO_2S$	121.16	122.1	59.3	20	44	1.84 ± 0.06	-

* For S_d calculation n=6.

Table S2. Precision and trueness data for the analysis of AA in spiked B-90 bread wheat sample.

Target Amino acid	Added (mg/100 g)	Repeatability (n=3)			Intermediate precision (n=3)		
		Found (mg/100 g)	Recovery (%)	RSD (%)	Found (m g/100 g)	Recovery (%)	RSD (%)
**Gly**	20.0	19.0	94.8	8.7	19.2	96.0	7.9
	40.0	43.9	109.8	2.9	44.5	111.2	6.6
**Ala**	20.0	19.9	99.6	6.2	20.2	100.8	4.8
	40.0	46.5	116.2	4.4	47.0	117.6	3.4
**Ser**	20.0	21.3	106.3	2.3	21.52	107.6	1.4
	40.0	40.5	101.3	6.5	41.04	102.6	3.5
**Pro**	20.0	20.1	100.7	7.2	20.4	101.9	3.2
	40.0	39.2	98.1	13.9	39.7	99.3	9.8
**Val**	20.0	20.1	100.3	2.9	20.3	101.5	3.6
	40.0	39.7	99.3	6.8	40.2	100.5	8.7
**Thr**	20.0	19.3	96.5	4.6	19.5	97.7	2.8
	40.0	41.9	104.7	6.6	42.4	106.0	6.7
**Asp**	20.0	20.6	103.2	0.9	20.9	104.5	2.7
	40.0	46.2	115.4	1.7	46.7	116.8	4.3
**Glu**	20.0	18.8	93.8	0.6	19.0	95.0	1.8

	40.0	35.6	88.9	4.8	36.0	90.0	7.2
**Ile**	20.0	19.4	97.0	2.3	19.6	98.2	5.2
	40.0	39.3	98.2	5.3	39.8	99.4	8.9
**Leu**	20.0	19.4	97.0	2.3	19.6	98.2	6.1
	40.0	48.7	121.8	2.8	49.3	123.3	5.4
**Gln**	20.0	18.9	94.4	6.5	19.1	95.6	5.6
	40.0	38.9	97.3	9.2	39.4	98.5	8.7
**Lys**	20.0	20.8	104.2	3.7	21.1	105.5	3.1
	40.0	34.3	85.7	5.6	34.7	86.8	13.7
**Met**	20.0	20.6	103.0	9.8	20.9	104.3	5.5
	40.0	48.1	120.2	5.7	48.7	121.7	7.6
**His**	20.0	18.8	93.9	1.8	19.0	95.1	2.3
	40.0	44.5	111.3	5.5	45.1	112.7	2.7
**Phe**	20.0	20.6	102.9	2.3	20.8	104.2	8.4
	40.0	40.1	100.2	11.1	40.6	101.4	11.3
**Arg**	20.0	19.1	95.3	7.3	19.3	96.5	5.6
	40.0	39.0	97.6	10.2	39.5	98.8	4.3
**Tyr**	20.0	20.4	102.0	4.0	20.7	103.3	3.8
	40.0	39.9	99.9	8.3	40.4	101.1	9.7
